# Rapid mimicry and emotional contagion in domestic dogs

**DOI:** 10.1098/rsos.150505

**Published:** 2015-12-23

**Authors:** Elisabetta Palagi, Velia Nicotra, Giada Cordoni

**Affiliations:** 1 Natural History Museum, University of Pisa, Pisa, Italy; 2 Unit of Cognitive Primatology and Primate Center, ISTC, CNR, Rome, Italy

**Keywords:** social play, mirroring response, empathy, emotional proximity, *Canis lupus familiaris*

## Abstract

Emotional contagion is a basic form of empathy that makes individuals able to experience others’ emotions. In human and non-human primates, emotional contagion can be linked to facial mimicry, an automatic and fast response (less than 1 s) in which individuals involuntary mimic others’ expressions. Here, we tested whether body (play bow, PBOW) and facial (relaxed open-mouth, ROM) rapid mimicry is present in domestic dogs (*Canis lupus familiaris*) during dyadic intraspecific play. During their free playful interactions, dogs showed a stronger and rapid mimicry response (less than 1 s) after perceiving PBOW and ROM (two signals typical of play in dogs) than after perceiving JUMP and BITE (two play patterns resembling PBOW and ROM in motor performance). Playful sessions punctuated by rapid mimicry lasted longer that those sessions punctuated only by signals. Moreover, the distribution of rapid mimicry was strongly affected by the familiarity linking the subjects involved: the stronger the social bonding, the higher the level of rapid mimicry. In conclusion, our results demonstrate the presence of rapid mimicry in dogs, the involvement of mimicry in sharing playful motivation and the social modulation of the phenomenon. All these findings concur in supporting the idea that a possible linkage between rapid mimicry and emotional contagion (a building-block of empathy) exists in dogs.

## 1. Introduction

Rapid mimicry is an involuntary, automatic and fast response (less than 1 s) through which individuals mimic others’ expressions [1]. This phenomenon is grounded in the automatic Perception–Action coupling of sensorimotor information that occurs in motor brain areas [2,3]. The discovery of mirror neurons in the premotor and parietal cortices of monkeys provided the neurophysiological evidence of this coupling [4–6]. This set of neurons fires when a monkey performs an action and when it observes a similar action performed by another individual [4].

Emotional contagion, a basic building-block of empathy, occurs when a subject shares the same affective state of another [1–3,7]. Although rapid mimicry and emotional contagion are distinct concepts, because each can occur without the other, they may interact with each other [7]. For instance, consolation [8–10] is driven by emotional contagion experienced by the consoler in response to victim anxiety, even without the involvement of mimicry. However, certain forms of emotional contagion can be mediated by mimicry [1–3]. In humans, the observation of facial expressions activates, similarly to monkeys, shared motor representations not only in premotor and parietal areas but also in insular and cingulate cortices, these latter being directly involved in processing visceromotor sensations: a sort of same face–same emotion process [11,12]. This process involves two steps. Firstly, the perception of others’ expressive behaviour automatically induces the observer to mimic such behaviour. Secondly, the mimicry of others’ behaviour can induce the observer to share the emotional state underpinning such behaviour [13]. From a behavioural perspective, humans show a higher latency to emotionally respond to others’ facial expressions when they are unable to mimic than when they are free to mimic the facial expressions perceived [14]. Moreover, measures of facial electromyography revealed congruent, greater and faster (less than 500 ms) facial muscle reactivity in human subjects classified as ‘highly empathic’ when they were exposed to happy and angry faces as stimuli. Accordingly, this highly empathic group of human subjects also reacted with a corresponding experience of emotion. By contrast, the group classified as ‘low empathic’ did not differentiate between happy and angry stimuli with either facial muscles or self-experience of emotion [15]. These findings support the hypothesis that rapid and automatically evoked facial mimicry may be one of the mechanisms for the occurrence of emotional contagion [15].

Rapid mimicry has been demonstrated also in non-human primates such as orangutans (*Pongo pygmaeus*) [16], chimpanzees (*Pan troglodytes*) [17] and geladas (*Theropithecus gelada*) [18]. During playful interactions of these species, rapid mimicry prolonged the interactions [17,18] and was highly expressed between subjects sharing close social bonds (e.g. mother–infant dyads) [18]. Moreover, in non-primate mammals, new lines of research are also revealing that in social rodents some forms of pro-social behaviour can be linked to the perception and discrimination of facial expressions and vocalizations [19–21].

As in many social species, in dogs signals combine body postures, including head and tail, and the expressive use of eyes, lips and teeth [22]. It has been shown recently that in dogs signals communicate their emotional states [23]. Dogs are able to follow others’ gaze, head and body orientation [24,25]. During their inter- and intraspecific playful interactions, dogs regularly express their positive emotional states via specific signals that are performed through both the face (relaxed open mouth (ROM); table 1) and the body (play bow (PBOW); table 1) [26,28,29].

**Table 1.** List and definitions of the play behavioural items used in the study.

play patterns used as control for the demonstration of rapid mimicry	
bite attempt/bite (BITE)	the dog opens its mouth and then attempts to bite or bites another individual
jump attempt (JUMP)	just before a dog jumps on another individual, it starts from a semi-crouching position (jump attempt) and then leaps away. During the jumping attempt, the dog crouches on its forelimbs and remains standing on its hind-legs for a while
play signals	
ROM	the mouth is relaxed and kept open at different gradients. The mouth can be opened (i) just a little revealing only the upper parts of the most forward teeth of the lower jaw and (ii) in a wider way completely revealing the lower and upper jaws [26]
PBOW	a dog crouches on its forelimbs, remains standing on its hind-legs, and may wag its tail and sometimes bark. The bow is a stable posture from which the animal can move easily in many directions, allows the individual to stretch its muscles and places the head of the bower below another animal in a non-threatening position [27]

Since dogs can discriminate emotional expressions of human faces [30] and body postures [31], respond to humans’ yawns [32,33], show basal levels of empathy [34] and react similarly to some emotional state changes of other dogs and humans [35,36], we expect that they are capable of sharing positive emotional states by reproducing expressions (ROM) and postures (PBOW) of conspecifics in a rapid and congruent way (rapid mimicry) during their playful interactions.

As emotions are commonly revealed via behavioural and somatic responses [37], it is probably adaptive for animals to discriminate others’ emotional expressions because this allows them to anticipate the behavioural response of the observed individual and to adjust their own behaviour accordingly [13,30]. In this view, if rapid mimicry is beneficial to the playmates, we expect that it enhances the success of the play sessions, measured by their duration over time.

In both humans and animals, mimicry is biased towards individuals who are more similar, familiar or socially close [2,38,39]. In dogs, there is recent evidence of a strong linkage between mutual gazing and dog–owner affiliation and that this linkage is mediated by oxytocin [40], which also plays a similar role in dog–dog social affiliation [41,42]. Since rapid mimicry passes through mutual gaze—an affiliation-driven behaviour—we expect that rapid mimicry is more frequent between dogs who share high level of affiliation and familiarity.

## 2. Methods

### 2.1 Data collection

In August 2012, we collected data in a dog-park located in a public green area in Palermo (Vincenzo Florio Junior, Sicily, Italy). We recorded each free play session of 49 pure-breed and mixed-breed domestic dogs (26 females and 23 males), who ranged from 3 to 72 months of age (mean 17.14±2.24 s.e.). With the permission of the owners, V.N. videotaped dogs daily from 18.00 until 21.00, collecting a total of 50 h of videos. We interviewed the owners to collect information on age, sex, pure/mixed breed and cohabitation with other dogs.

A play session began when one partner directed a playful pattern towards a conspecific who responded with another playful pattern. A session ended when playmates ceased their activities, one of them moved away or when a third individual interfered, thus interrupting the interaction. If another play session began after a delay of 10 s, that session was counted as new [18]. Play sessions always began and ended spontaneously, because the owners were asked to never interfere or try to interrupt the playful session. However, it is worth noting that we never observed any playful session escalating into serious aggression. Obviously, if any aggression occurred outside a playful context, the owner could freely interrupt the conflict.

For each play session analysed, we recorded: (i) identities of the subjects (i.e. name, sex, age and breed), (ii) playful patterns and their exact sequence and time (table 1), (iii) length (s) of interaction, and (iv) kind of social relationship shared by the interacting animals (see below).

The kind of social relationships shared by dogs within each dyad was determined by interviewing each owner separately. We divided the quality of the social bond into three classes: ‘friends’ (the dogs that lived together or regularly exchanged playful, affiliative and aggressive interactions—also exploring the environment together—at least three times per week); ‘acquaintances’ (the dogs that met together and exchanged playful, affiliative and aggressive interactions—also exploring the environment together—no more than twice a month); and ‘strangers’ (dogs that have never interacted before). We found a total agreement between the categorizations given by each owner in defining the relationships of their dogs.

### 2.2 Data analysis, operational definitions and statistics

Video-analysis was conducted using VLC 2.1.5 Rincewind software by V.N. with an accuracy of 0.4 s. Inter-observer reliability was tested by G.C. and E.P. The Cohen’s *κ* values obtained for the behavioural items described in table 1 were always more than 0.85.

To examine the presence of Rapid Mimicry, defined as the mirror response given by the observer within 1 s from the perception of the stimulus [16], we focused on two specific playful signals: PBOW and ROM (table 1). To check for the presence of the phenomenon of Rapid Mimicry both for PBOW and ROM, we selected two behavioural patterns—JUMP and BITE, respectively—that are performed via motor actions which strongly overlap with those of the two signals considered (table 1). We measured the displays of one individual (the observer, hereafter) to see whether they varied as a function of the signal displayed by the playmate (the signaller, hereafter) within a 1 s time window. The signaller was defined as the first playmate who emitted a stimulus (PBOW and ROM; JUMP and BITE). To reliably assess that the response produced by the observer was actually elicited by the stimulus emitted by the signaller, we considered only those interactions in which the observer looked at the signaller and did not show either ROM or PBOW in the 1 s prior to the emission of the stimulus by the signaller. Via exact Wilcoxon paired sample test corrected for ties [43], we compared the frequency of congruent response (ROM_observer_/ROM_signaller_) and the frequency of incongruent response (ROM_observer_/BITE_signaller_). We did the same comparison for PBOW and JUMP (PBOW_receiver_/PBOW_signaller_ versus PBOW_receiver_/JUMP_signaller_).

In addition, we also introduced a further control to ascertain that the congruence response is actually linked to the presence of the signals ROM and PBOW and does not characterize the motor patterns which strongly resemble them in the motor performance (BITE and JUMP). Hence, via exact Wilcoxon paired sample test, we compared the frequency of potential congruent response (BITE_observer_/BITE_signaller_) and the frequency of incongruent response (BITE_observer_/ROM_signaller_). We carried out the same comparison for JUMP and PBOW (JUMP_receiver_/JUMP_signaller_ versus JUMP_receiver_/PBOW_signaller_).

In the case of normal distributions we employed parametric statistics, otherwise non-parametric statistics was used.

We evaluated which factors could explain the variation of body–facial rapid mimicry (electronic supplementary material, table S1), via a generalized linear mixed model analysis (GLMM). Body–facial rapid mimicry was the dependent variable, which was normally distributed (Anderson-Darling=1.9743, n.s.; EasyFit 5.5 Professional). The fixed factors and the random variables are reported in table 2. In order to be conservative as much as possible, we used robust estimation to handle violations of model assumptions. We tested models for each combination involving the variables of interest (table 2), spanning from a single-variable model to a model including all the fixed factors (full model). To select the best model, we used the Akaike’s corrected information criterion (AICc), which corrects the Akaike’s information criterion (AIC) for small sample sizes. The model with the lowest value of AIC was considered to be the best model.

**Table 2.** Description of the variables used in GLMM analyses of body–facial rapid mimicry (PBOW, play bow; ROM, relaxed open mouth).

name	type
*dependent variables*	
body-facial rapid mimicry	continuous
*fixed explanatory variables*	
individual characteristics	
sex player 1	nominal (0=male; 1=female)
sex player 2	nominal (0=male; 1=female)
age player 1	ordinal (0=0–3 months; 1=4–6 months; 2=7–12 months; 3=13–24 months; 4=>24 months)
age player 2	ordinal (0=0–3 months; 1=4–6 months; 2=7–12 months; 3=13–24 months; 4=>24 months)
size player 1 (length, excluding tail)	ordinal (0=<50 cm; 1=50–100 cm; 2=>100 cm)
size player 2 (length, excluding tail)	ordinal (0=<50 cm; 1=50–100 cm; 2=>100 cm)
cohabitating with other dog/s	nominal (0=no; 1=yes)
breed/mixed race	nominal (0=breed; 1=mixed)
relationship quality	
social bonding	ordinal (1=friends; 2=acquaintances; 3=strangers)
*random variables*	
player 1 identity	nominal
player 2 identity	nominal
frequency of play signals perceived (PBOW *plus* ROM)	continuous

## 3. Results

**Figure 1.**
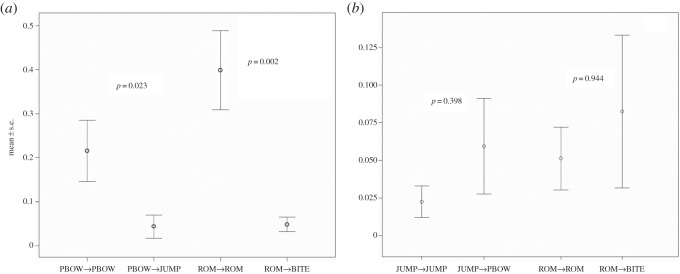
(*a*) Number of responses per stimulus perceived: PBOW–PBOW, congruent; PBOW–JUMP, incongruent; ROM–ROM, congruent; ROM–BITE, incongruent. (*b*) Number of responses per stimulus perceived: JUMP–JUMP, congruent; JUMP–PBOW, incongruent; BITE–BITE, congruent; BITE–ROM, incongruent.

We demonstrated the presence of both body (PBOW) and facial (ROM) Rapid Mimicry in dogs. The receiver performed a PBOW more frequently in the first second after perceiving a PBOW (congruent response) than after perceiving a JUMP (incongruent response) (PBOW_receiver_/PBOW_signaller_ versus PBOW_receiver_/JUMP_signaller_: Wilcoxon *T*=10.00, *n*=21, ties=8, *p*=0.023; figure 1*a*). No significant difference was present between JUMP_receiver_/JUMP_signaller_ and JUMP_receiver_/PBOW_signaller_ (Wilcoxon *T*=9.00, *n*=21, ties=14, *p*=0.398; figure 1*b*). The receiver performed a ROM more frequently in the first second after perceiving a ROM (congruent response) than after perceiving a BITE (incongruent response) (ROM_receiver_/ROM_signaller_ versus ROM_receiver_/BITE_signaller_: *T*=2.00, *n*=21, *p*=0.002; figure 1*a*). No significant difference was present between BITE_receiver_/BITE_signaller_ and BITE_receiver_/ROM_signaller_ (Wilcoxon *T*=17.50, *n*=21, ties=13, *p*=0.944; figure 1*b*). Owing to the high individual variability in ROM and PBOW performance and in order to be conservative as much as possible, we restricted all these analyses only to the subjects (*n*=21) who perceived at least two stimulus events per each condition.

**Figure 2.**
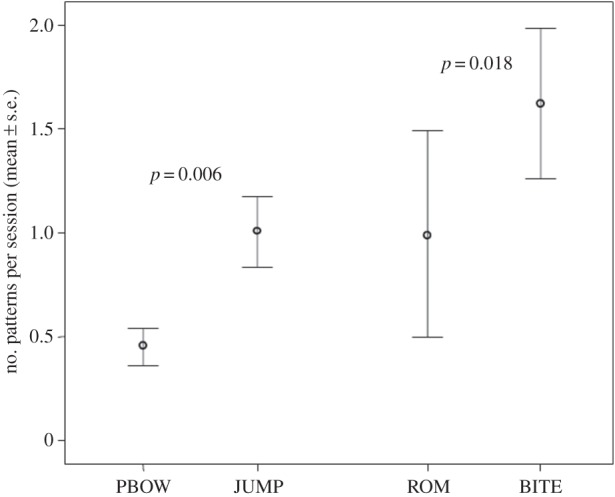
Mean (±s.e.) frequency of PBOW, JUMP, ROM, BITE (number of patterns per play session) performed by dogs.

If certain behaviours occur more often than others the possibility that they can produce a higher level of congruence exists. Hence, to rule out such possibility we compared the mean frequency of PBOW and JUMP and of ROM and BITE performed by each dog (number per play session). We found that JUMP and BITE were significantly more frequent than PBOW and ROM, respectively (JUMP versus PBOW; Wilcoxon *T*=31.50, *n*=21, ties=1, *p*=0.006; BITE versus ROM; Wilcoxon *T*=36.00, *n*=21, ties=2, *p*=0.018; figure 2).

**Figure 3.**
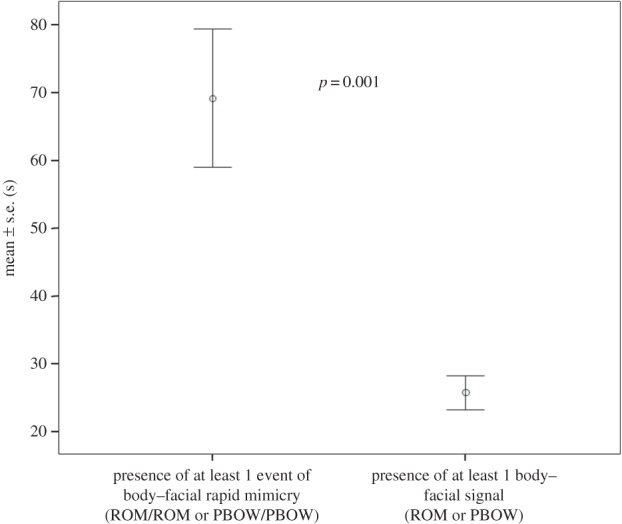
Mean (±s.e.) duration of the play session as a function of the presence of at least one event of body–facial rapid mimicry (ROM/ROM or PBOW/PBOW) and the absence of body–facial rapid mimicry but the presence of at least one body–facial signal (ROM or PBOW).

Play interactions characterized by at least one event of body and facial rapid mimicry (congruent response) were significantly longer than those interactions characterized by the mere presence of one signal (ROM or PBOW) which was not followed by any event of mimicry (incongruent response) (paired-sample *t*-test; *t*=2.586, d.f.=11, *p*=0.025; figure 3).

**Figure 4.**
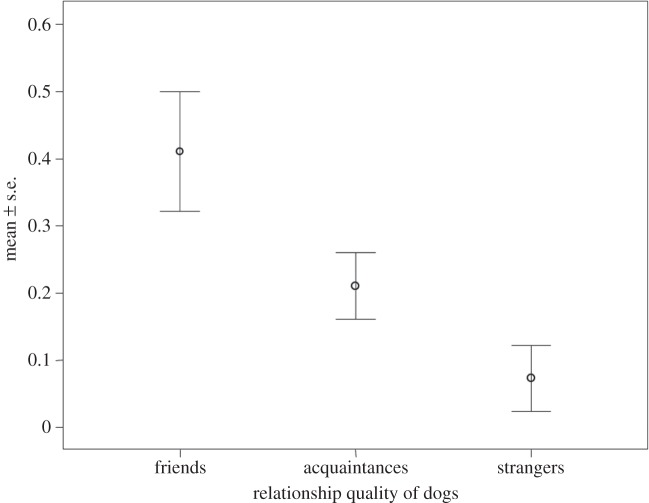
Frequency of body–facial rapid mimicry as a function of social bonding.

To verify which factors affected the frequency of body–facial rapid mimicry, we ran a GLMM that included only those dyads characterized by at least two stimulus events. We found that only social bonding remained in the best model (AICc=12.088; table 3) with ‘friends’ showing the highest levels of body–facial rapid mimicry (figure 4). None of the random variables (identity of players, play signals perceived; table 2) had any effect on the distribution of body–facial rapid mimicry. The full model was the worst (AICc=37.790).

**Table 3.** Best GLMM explaining the distribution of body–facial rapid mimicry (AIC_c_=12.088). (d.f., degrees of freedom; PBOW, play bow; ROM, relaxed open mouth).

body–facial rapid mimicry	d.f.1	d.f.2	*F*	significance level
fixed factors				
social bonding	2	36	6.018	*p*=0.006

	estimate	s.e.	*Z*	sig.
random factors				
player 1 identity	0.00^*a*^			
player 2 identity	0.00^*a*^			
frequency of play signals (PBOW *plus* ROM)	0.00	0.00	0.121	0.903

^*a*^This parameter is redundant.

## 4. Discussion

Matching one’s own behaviour with that of others gives individuals the possibility to appropriately react and synchronize their activity with those of group members [2]. The context of play, due to its positive, emotional involvement, provides a good substrate to investigate the phenomenon of rapid mimicry [44]. Experiencing others’ emotional states instantly allows an individual to foresee their playmates’ intentions and fine-tune motor sequences accordingly [45]. Our findings reveal that rapid mimicry occurs not only in humans and other primate species but also in dogs under the playful context. A high level of rapid mimicry was recorded with a mean of 77% of dogs that congruently reacted after perceiving PBOWs and ROMs. Interestingly, a similar percentage (72%) was also found for yawn contagion (an empathy-related phenomenon) in dogs when infected by humans [33].

Rapid mimicry facilitates communicative exchanges and behavioural coordination in the sequence of actions [17,45]. The coordination of emotional signals between subjects is evolutionarily stable only when the emission of the signal and the response associated with it are beneficial for both the sender and the receiver [13,46]. In our dogs, the playful sessions characterized by rapid mimicry lasted longer than those characterized by incongruent response (figure 3). Being involved in prolonged interaction is advantageous for the playmates, who increase the opportunity to assess their reciprocal ability and to test their social relationships [44]. Even though we cannot disentangle the cause–effect relationship between rapid mimicry and the real emotional engagement to play, we demonstrated that in dogs the two phenomena are inter-connected and that a positive affective state is shared. It is worth noting, however, that in non-human primates behavioural mimicry not only occurs within pre-existing bonds but also modulates such bonds. In an elegant experiment, Paukner *et al*. [47] demonstrated that capuchin monkeys preferentially directed their affiliative behaviours towards an experimenter who imitated them with respect to an experimenter who performed contingent actions without imitation. Thus, mimicry seems to modulate the social attitude between the two interacting subjects.

In our dogs, the socio-emotional factors influenced the propensity of individual mimicry as it occurs in humans [1] and non-human primates [18,48]. One of the most recent hypotheses, *the Emotion Mimicry in Context*, predicts that human subjects mimic an emotion only if they share the perspective that gave rise to that specific emotion. In particular, this hypothesis focuses on the relationship between sender and receiver and suggests that emotional sharing can be mediated by rapid mimicry and depends on the quality of this relationship (the context) and functions as a social regulator [49]. In dogs, the GLMM showed that only social bonding predicted the distribution of rapid mimicry, whose frequency was greatest in response to friends, then acquaintances, and lastly strangers (figure 4). The effect of familiarity on mimicry indicates that positive affect may regulate mimicry in the domestic dog. Interestingly, the same ‘empathic gradient’ was also reported in humans, whose levels of yawn contagion [39] and facial mimicry [49,50] were strongly affected by the familiarity and intimacy shared by the subjects involved. Hence, also in dogs it seems that rapid mimicry is based on a ‘motor’ and an ‘affective’ identification [13,51,52]: the more familiar and socially close two dogs are, the easier the identification with the playmate. The fact that social closeness predicts rapid mimicry in dogs is consistent with the hypothesis that this phenomenon is a possible means to activate shared representations, as occurs for yawn contagion between dogs and humans [33] and between wolves [41]. In experimental work, Muller *et al.* [30] found that through experience gained by social interactions with their owners, dogs are able to form a huge variety of memories of human facial expressions that goes beyond the purely perceptual level. The authors suggested that the ability to finely discriminate facial expressions also implies the possibility that dogs are able to catch the emotional meaning underpinning such specific facial expressions. Moreover, the importance has been underlined recently of dog–human mutual gazing in increasing oxytocin concentration in the interactive subjects and consequently their social affiliation [40,41,53]. Mimicry, based on and facilitated by mutual gaze [50,54], could have a direct function in this emotional positive loop by connecting the dogs and fostering their social attachment.

In conclusion, our results demonstrate the presence of rapid mimicry in dogs (for PBOW and ROM), the involvement of mimicry in sharing and increasing playful motivation (measured by the duration of the playful session) and the social modulation of this phenomenon (effect of the relationship quality shared by subjects). All these findings concur in supporting the idea that a possible linkage between rapid mimicry and emotional contagion (a basic form of empathy) exists also in dogs. Further research should focus on the demonstration of rapid mimicry in wolves to evaluate if the phenomenon in dogs has been shaped by the domestication process or is evolutionarily rooted in the line of social carnivores.

## Data accessibility

The data used for this study are provided as the electronic supplementary material to this article. The statistical tools Easyfit and SPSS used in this study are proprietary software. However, trials (limited time/limited functionality) can be found online on the producer’s websites (EasyFit: www.mathwave.com; SPSS: https://www-01.ibm.com).
